# Diagnostic Potential of Cell-Free and Exosomal MicroRNAs in the Identification of Patients with High-Risk Colorectal Adenomas

**DOI:** 10.1371/journal.pone.0160722

**Published:** 2016-10-19

**Authors:** Ryo Uratani, Yuji Toiyama, Takahito Kitajima, Mikio Kawamura, Junichiro Hiro, Minako Kobayashi, Koji Tanaka, Yasuhiro Inoue, Yasuhiko Mohri, Takao Mori, Toshio Kato, Ajay Goel, Masato Kusunoki

**Affiliations:** 1 Department of Gastrointestinal and Pediatric Surgery, Division of Reparative Medicine, Institute of Life Sciences, Graduate School of Medicine, Mie University, Mie 514–8507, Japan; 2 Moriei Hospital, Kuwana city, Mie 511–0038, Japan; 3 Tohyama Hospital, Tsu city, Mie 514–0043, Japan; 4 Center for Gastrointestinal Research & Center for Epigenetics, Cancer Prevention and Cancer Genomics, Baylor Research Institute and Charles A Sammons Cancer Center, Baylor University Medical Center, Dallas, Texas, 75246–2017, United States of America; Saint Louis University, UNITED STATES

## Abstract

**Background:**

Although there is a growing interest in developing circulating microRNA (miRNA) as noninvasive diagnostic biomarkers for the detection of high-risk colorectal adenomas and early-stage CRCs, but the comparative diagnostic significance of serum vs. exosomal miRNAs remains unexplored.

**Methods:**

Based upon published literature, we performed an initial discovery step by investigating the expression of a miRNA panel in 20 normal colonic mucosa, 27 adenomas, and 19 CRC tissues. We performed subsequent validation by quantifying expression of candidate miRNAs in total serum and in exosomes from 26 adenoma patients and 47 healthy controls, and evaluated their clinical significance and potential diagnostic value in colorectal adenomas.

**Results:**

We observed that the expression of four miRNAs, miR-21, miR-29a, miR-92a, and miR-135b, was significantly higher in colorectal adenomas vs. normal colonic mucosa. During validation, expression of miR-21, miR-29a and miR-92a in serum was significantly higher in adenomas vs. healthy controls, significantly correlated with adenoma size and total adenoma number within the colorectum, and significantly discriminated patients with advanced adenomas. In contrast, although exosomal miR-21 and miR-29a levels in adenoma patients were significantly higher than those of healthy volunteers, only exosomal miR-21 significantly correlated with adenoma size and total adenoma number, and could discriminate patients with high-risk adenomas.

**Conclusion:**

Compared to exosomal miRNAs, serum levels of miR-21, miR-29a and miR-92a are superior diagnostic biomarkers in patients with high-risk adenomatous polyps.

## Introduction

Despite the known benefits of early screening and treatment, colorectal cancer (CRC) remains the second most common cause of cancer-related deaths in the United States [1], and the fourth most deadliest cancer worldwide [2]. The large majority of CRCs are thought to arise from adenomatous polyps within the colorectum. This fact has also been proven in the case of hereditary colon cancer syndromes, such as familial adenomatous polyposis, in which patients are at absolute risk for developing colorectal carcinoma [3]. Within the pool of colorectal adenomas, large or advanced adenomas (≥1 cm) are believed to be the true precursors and major targets for any CRC screening program, as these progress at a rate of ~1% per year [4].

Currently, CRC screening modalities comprise of fecal occult blood tests (FOBT), sigmoidoscopy, and colonoscopy [5]. In many resource-limited countries, FOBT-based screening remains the primary option for CRC screening, in spite of its insufficient sensitivity [6, 7]. Moreover, sigmoidoscopy and colonoscopy are invasive, expensive and are highly dependent on the expertise of the gastroenterologist to detect cancerous and precancerous lesions. In fact, up to 12% of precancerous lesions miss detection, either as a result of the polyp morphology (so-called “flat” or “serrated” polyps) or due to failure to visualize the entire colon [8], and approximately 10% of CRCs occur in individuals within 3 years of a screening colonoscopy [9]. Therefore, more affordable, minimally invasive and non-invasive approaches are much needed to improve the overall sensitivity and patient compliance for CRC screening.

MicroRNAs (miRNAs) are 18–22 nucleotide non-coding RNAs that post-transcriptionally regulate gene expression and control various cellular mechanisms including tumorigenesis and the development of various types of cancers [10–12]. Recent studies have shown that there are abundant load of circulating miRNAs in the systemic circulation. These circulating miRNAs remain stable in unfavorable physiological conditions such as boiling, very low or high pH levels, extended storage time, and repeated freeze-thaw cycles [11, 13].

Because serum and plasma are relatively easy to obtain, circulating miRNAs are emerging as one of the most promising substrates for diagnostic biomarker development in cancer [14]. Many recent studies have shown that the expression patterns of serum miRNAs can potentially identify various types of cancer, including lung, prostate, breast, ovarian, and liver cancer [12, 15]. In CRC, accumulating evidence firmly supports the existence of unique “miRNA signatures” in biological fluids that can facilitate earlier detection of the tumor and also assist in predicting disease recurrence and therapeutic outcome for currently available treatment regimens [16]. We also recently demonstrated that miR-21 in serum is a promising biomarker for the early detection and prognosis of CRC [17]. Additionally, we reported that serum miR-200c and miR-203, which are epithelial-to-mesenchymal transition-related miRNAs, has strong potential to serve as a non-invasive biomarker for CRC prognosis and to predict metastasis [18, 19].

Exosomes are small lipid-bilayer enclosed extracellular vesicles and have the capacity to envelop specific miRNAs [20]. Thus, exosomes maintain the integrity of their cellular contents in circulation [21–23]. Based upon this paradigm, the exosomal contents that reflect the features of their donor cells (cancer cells) can be transported to other recipient cells within the tumor microenvironment or distant organ [24–26]. Therefore, these evidences suggested that specific exosomal-miRNAs are related to the unique cancer phenotypes and may function as meaningful diagnostic biomarkers [20, 25, 27, 28]. However, there are no data available to directly assess whether circulating vs. exosomal miRNAs may be more robust biomarkers, with regards to sensitivity and specificity, for the early detection of colorectal neoplasia.

In the present study, to further strengthen the clinical application of circulating miRNAs for the diagnosis of early-stage colorectal neoplasia (advanced adenomas and early-stage cancers), we first performed a discovery step based upon miRNAs with published evidence as potential diagnostic biomarkers of CRC. Next, we selected candidate miRNAs based upon their expression in tissues according to the normal colonic mucosa, adenoma, and carcinoma developmental sequence. Thereafter we identified miRNAs that were overexpressed in adenoma tissues vs. normal colonic mucosa, and validated their expression levels in serum and within the exosomes, from patients with adenomas and healthy controls. Furthermore, through statistical analysis, we evaluated the clinical significance of these miRNAs in patients with colonic adenomas, particularly with respect to their diagnostic potential. Finally, we determined the diagnostic accuracy and robustness of serum vs. exosomal candidate miRNAs in identification of patients with high-risk adenomas, who are deemed to be an ideal target population for any CRC screening strategy.

## Materials and Methods

### Study design

In total, 139 colorectal specimens were obtained at Mie University Medical Hospital, Moriei Hospital, and Tohyama Hospital, Japan between January 1, 2013 and December 31, 2013. This was a three-phase study, which aimed to discover, validate, and determine the potential contribution of serum miRNAs in colonic adenoma patients. During the initial discovery phase, we performed a careful literature review to search for candidate miRNAs that were reported to be overexpressed in both cancer tissue and in the circulation of CRC patients, and selected four miRNAs (miR-21, miR-29a, miR-92a, and miR-135b) for further analysis **(S1 Table)**. In the second phase, we investigated whether these four candidate miRNAs from the initial discovery step were dysregulated in the colorectal adenoma–carcinoma sequence using 66 tissues from 20 colonic mucosae, 27 adenomas, and 19 CRCs. The final phase aimed to validate the biological significance and determine the translation of these candidate miRNAs into clinically useful, non-invasive serum biomarkers for the detection of adenoma patients. In this regard, serum samples were collected from 74 outpatients before total colonoscopy. One gastroenterologist performed colonoscopy and diagnosed 26 adenoma patients and 47 healthy subjects. The **S2 Table** and **Table 1**describe in detail the clinic-pathological characteristics of the adenoma patients and healthy controls. Each serum specimen was divided into two parts for the extraction of miRNAs in total serum and within the exosomal fraction, respectively **(S1 Fig)**.

**Table 1:** Clinicopathological findings in patients with colonic polyps.

Patients	Sex	Age	Maximum Diameter	Adenoma Counts	Pathological Findings
1	M	71	7	1	tubular adenoma
2	F	66	7	2	mild hyperplastic/mild hyperplastic
3	F	70	5	2	tubular adenoma
4	F	64	6	1	tubular adenoma
5	M	64	10	2	tubular adenoma/tubular adenoma
6	M	77	10	3	tubular adenoma/tubular adenoma/tubular adenoma
7	M	75	10	4	serrated/serrated/tubular adenoma/tubular adenoma
8	F	70	10	1	tubular adenoma
9	M	72	10	1	serrated
10	F	48	4	1	no data
11	M	79	3	1	no data
12	F	80	7	1	tubular adenoma
13	M	64	10	5	hyperplastic/serrated/hyperplastic/tubular adenoma
14	F	62	7	1	serrated
15	M	62	7	1	tubular adenoma
16	F	50	15	1	tubular adenoma
17	F	62	7	1	serrated
18	M	85	7	1	no data
19	M	76	7	2	serrated/tubular
20	M	80	5	1	tubular adenoma
21	F	29	3	3	no data
22	F	53	7	1	tubular adenoma
23	M	65	3	1	no data
24	M	65	7	3	tubular adenoma/metaplastic/ tubular adenoma
25	M	66	4	1	no data
26	M	66	7	1	tubular adenoma

### Ethics statement

Both tissue- and serum-based specimen collection and studies were approved by the institutional review boards of Mie University Hospital, Moriei Hospital and Tohyama Hospital, Japan. All participants provided written informed consent and indicated willingness to donate their blood and tissue samples for research.

### RNA isolation and qRT-PCR from formalin-fixed paraffin-embedded (FFPE) tissues

Total RNA was isolated from FFPE samples using the RecoverAll Total Nucleic Acid Isolation Kit (Ambion Inc., Austin, TX, USA). Briefly, tissue sections were microdissected to enrich for neoplastic cells, followed by deparaffinization and RNA extraction according to the manufacturer’s protocol. Total RNA was eluted in the appropriate buffer, and quantified using a NanoDrop Spectrophotometer (NanoDrop Technologies, Wilmington, DE, USA). Reverse-transcription (RT) reactions were carried out using the TaqMan MicroRNA Reverse Transcription Kit (Applied Biosystems, Foster City, CA, USA) in a total reaction volume of 15 μL. MiR-21, miR-29a, miR-92a, miR-135b, and miR-16 were quantified in duplicate by quantitative RT-polymerase chain reaction (qRT-PCR), using MicroRNA Assay Kits (Applied Biosystems, Foster City, CA, USA).

qRT-PCR was performed on the StepOne Real Time PCR System (Applied Biosystems, Foster City, CA, USA) using the following cycling conditions: 95°C for 10 min, followed by 45 cycles of 95°C for 15 s, and 60°C for 1 min.

### RNA isolation and qRT-PCR from total serum

RNA extraction and miRNA enrichment from all sera were performed using the Qiagen miRNeasy Kit (Qiagen, Valencia, CA, USA). Briefly, 250 μL of serum was thawed on ice and centrifuged at 10,000 rpm for 5 min to remove cell debris. Next, the same amount of starting material (200 μL) from the supernatant was lysed in five volumes of Qiazol solution. To normalize any inadvertent sample-to-sample variations during the RNA isolation procedure, RT, and PCR, 25 fmol of synthetic *Caenorhabditis elegans* miRNA (cel-*miR-39*) was added to each denatured sample. Small RNAs were then enriched and purified following the manufacturer’s protocol, with the exception that the enriched small RNAs were eluted in 40 μL of nuclease-free water. For miRNA-based RT-PCR assays, 2 μL of enriched small RNAs from serum were reverse transcribed using the TaqMan MicroRNA Reverse Transcription Kit (Applied Biosystems, San Diego, CA, USA) in a total volume of 5 μL. PCR reactions for quantification of miR-21, miR-29a, miR-92a, miR-135b, and cel-miR-39 were performed in duplicate using TaqMan 2× Universal PCR Master Mix according to the manufacturer’s protocol.

### Isolation of exosomes from serum, exosomal miRNA extraction and qRT-PCR

The exosomes were isolated from all sera using ExoQuick™ kit (System Biosciences, EXOQ20A-1) according to the manufacturer’s protocol. Briefly, all serum samples were thawed and centrifuged at 10,000 rpm for 5 min to remove cells and cell debris. Next, 250 μL of the supernatant was mixed with 63 μL of ExoQuick™ solution and incubated at 4°C for 30 minutes. Thereafter, the mixture was centrifuged at 1500 g for 30 minutes, and the acquired exosome pellet was resuspended in 20 μL nuclease-free water. Isolated exosomes from all serum using ExoQuick™ kit were well characterized by exosomal protein markers (CD63, CD9 and CD81) and not specific exosomes derived from colonic tissues. Extraction of miRNAs from exosomes was performed using the Qiagen miRNeasy Kit (Qiagen, Valencia, CA, USA) according to the manufacturer’s protocol. Briefly, exosomes purified from 250 μL of serum were diluted with 500 μL of Qiazol Lysis Reagent (Qiagen). After 5 min of incubation at room temperature, 5 μL of 5 nM syn-cel-miR-39 miScript miRNA Mimic and 100μL chloroform were added to each sample followed by vortexing for 15 s. Subsequent extraction and cartridge work were carried out according to the manufacturer’s protocol, with the exception that the final RNA elution was performed with 40 μL of RNase-free water. For miRNA-based RT-PCR assays, 2 μL of enriched small RNAs from serum were reverse transcribed using the TaqMan MicroRNA Reverse Transcription Kit (Applied Biosystems, San Diego, CA, USA) in a total volume of 15 μL. PCR reactions for quantification of miR-21, miR-29a, miR-92a, miR-135b, and cel-miR-39 were performed in duplicate using TaqMan 2× Universal PCR Master Mix according to the manufacturer’s protocol.

### Calculation of miRNA expression

Expression levels of serum or tissue miRNAs were normalized using cel-*miR-39* (for serum samples) and *miR-16* (for FFPE tissue samples) using the 2^–ΔCt^ method. Differences between the groups are presented as ΔCt, indicating differences between Ct values of miRNAs of interest and Ct values of normalizer miRNAs.

### Statistical analysis

Results were expressed as mean ± standard deviation. Mann–Whitney U and Kruskal–Wallis analyses of variance were used to evaluate statistical differences in serum or tissue miRNA expression between unpaired groups and multiple comparison groups, respectively. The Spearman’s correlation test was used to examine correlations between miRNA expression in serum and adenoma size and number, and between miRNA expression in serum and exosomes. Receiver operating characteristic (ROC) analysis was performed to determine the diagnostic performance of miRNAs expression levels in distinguishing patients with colorectal adenomas from healthy control subjects. Sensitivity against one minus specificity was plotted at each cutoff threshold, and the area under the curve (AUC) values that reflected the probability of correctly identifying adenoma patients from control subjects were computed. The optimal cutoff thresholds for diagnosis were obtained using the Youden’s index (30). In brief, the optimal cutoff threshold values were determined at the point on the ROC curve at which the Youden’s index (sensitivity + specificity– 1) was maximum. A multivariable logistic regression model was used to calculate odds ratios (ORs) for age- and sex-adjusted cases associated with adenoma according to serum miRNA levels. All *P* values were two-sided, and those less than 0.05 were considered statistically significant. All statistical analyses were carried out using Medcalc 12.3 for Windows (Broekstraat 52, 9030, Mariakerke, Belgium).

## Results

### MiR-21, miR-29a, miR-92a, and miR-135b tissue levels increased according to tumor progression (normal-adenoma-carcinoma sequence)

We first investigated miR-21, miR-29a, miR-92a, and miR-135b expression in normal colonic mucosa (NCM), colonic adenomas, and CRC by qRT-PCR to determine changes in expression across the colorectal normal-adenoma-carcinoma sequence.

**Fig 1:**
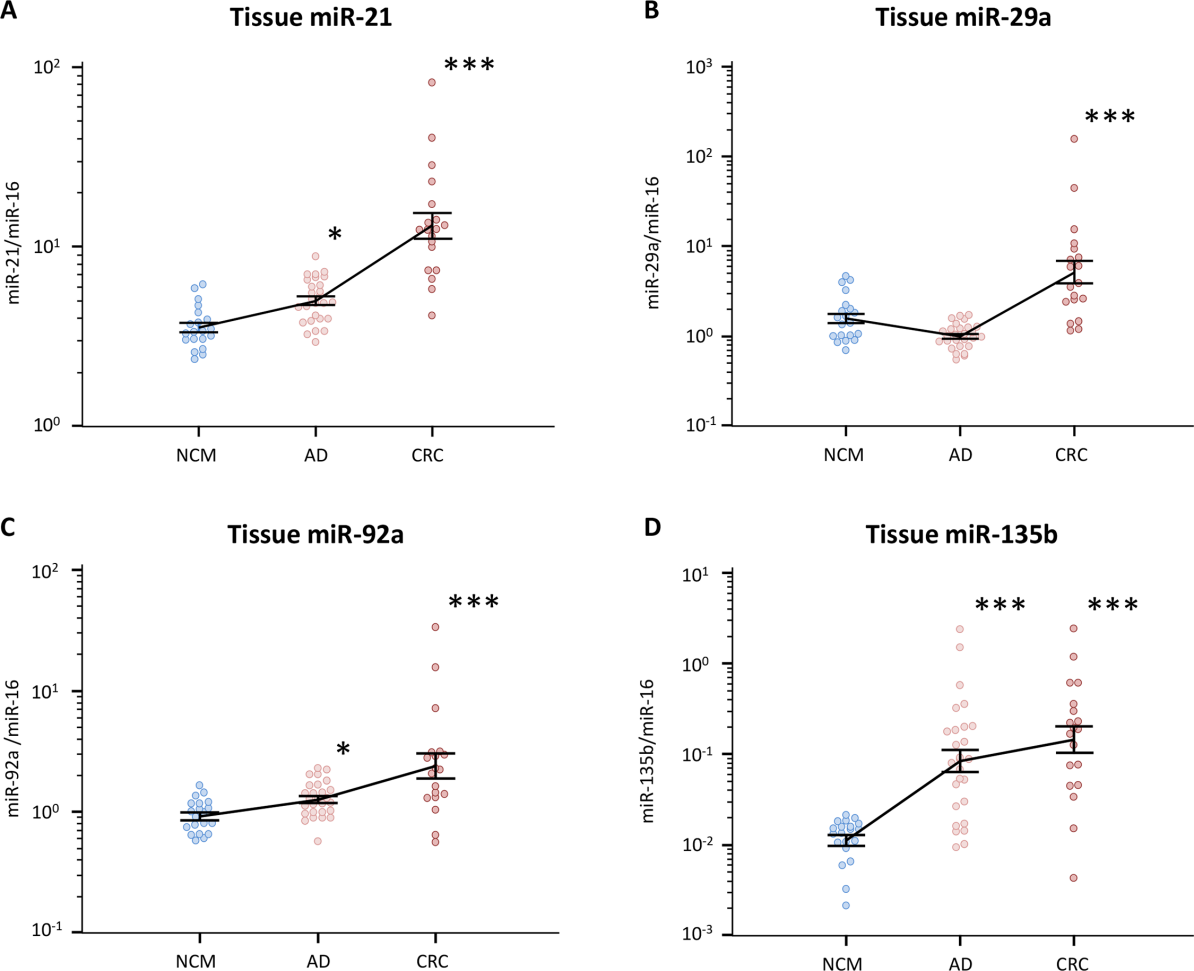
MiRNAs expression levels in tissue specimens during normal-adenoma-carcinoma sequence. **(A)** miR-21, **(B)** miR-29a, **(C)** miR-92a, and **(D)** miR-135b. The y-axis (log10 scale) represents relative expression of miRNAs normalized to miR-16 in normal colonic mucosa (NCM; n = 20), adenomatous polyps (AD; n = 27) and colorectal cancer (CRC; n = 19) tissues. All assays were carried out in duplicate. Data (means ± standard deviation) were analyzed by Mann–Whitney U and Kruskal–Wallis analyses for multiple comparisons to confirm a difference between groups (*P<0.05; ***P<0.001).

We found that miR-21, miR-29a, miR-92a, and miR-135b levels were significantly higher in CRC tissues compared with NCM **(Fig 1)**. Additionally, the expression of three of the four microRNAs (miR-21, miR-92a, and miR-135b) significantly increased in a stepwise manner during normal-adenomatous polyps-malignant colorectal cancer, and their expression levels in adenomatous polyps were significantly elevated compared with NCM **(Fig 1)**. Thus, miR-21, miR-92a, and miR-135b may therefore be promising non-invasive diagnostic biomarkers for identifying pre-malignant lesions in the colorectum, even when detected in the circulation.

### Characteristics of adenoma patients and healthy controls

The clinical characteristics of adenoma patients and healthy controls included in the study are summarized in **S2 Table**. No statistically significant differences were observed for age between healthy subjects (63±16.3 years) and patients with adenomas (66±11.8 years) (*P* = 0.79). Likewise, there were no gender differences between the different groups, with 23 men and 24 women in the control group, and 15 men and 11 women in the adenoma group (*P* = 0.63).

### Expression levels of serum miR-21, miR-29a and miR-92a in adenoma patients are significantly higher than in healthy volunteers

**Fig 2:**
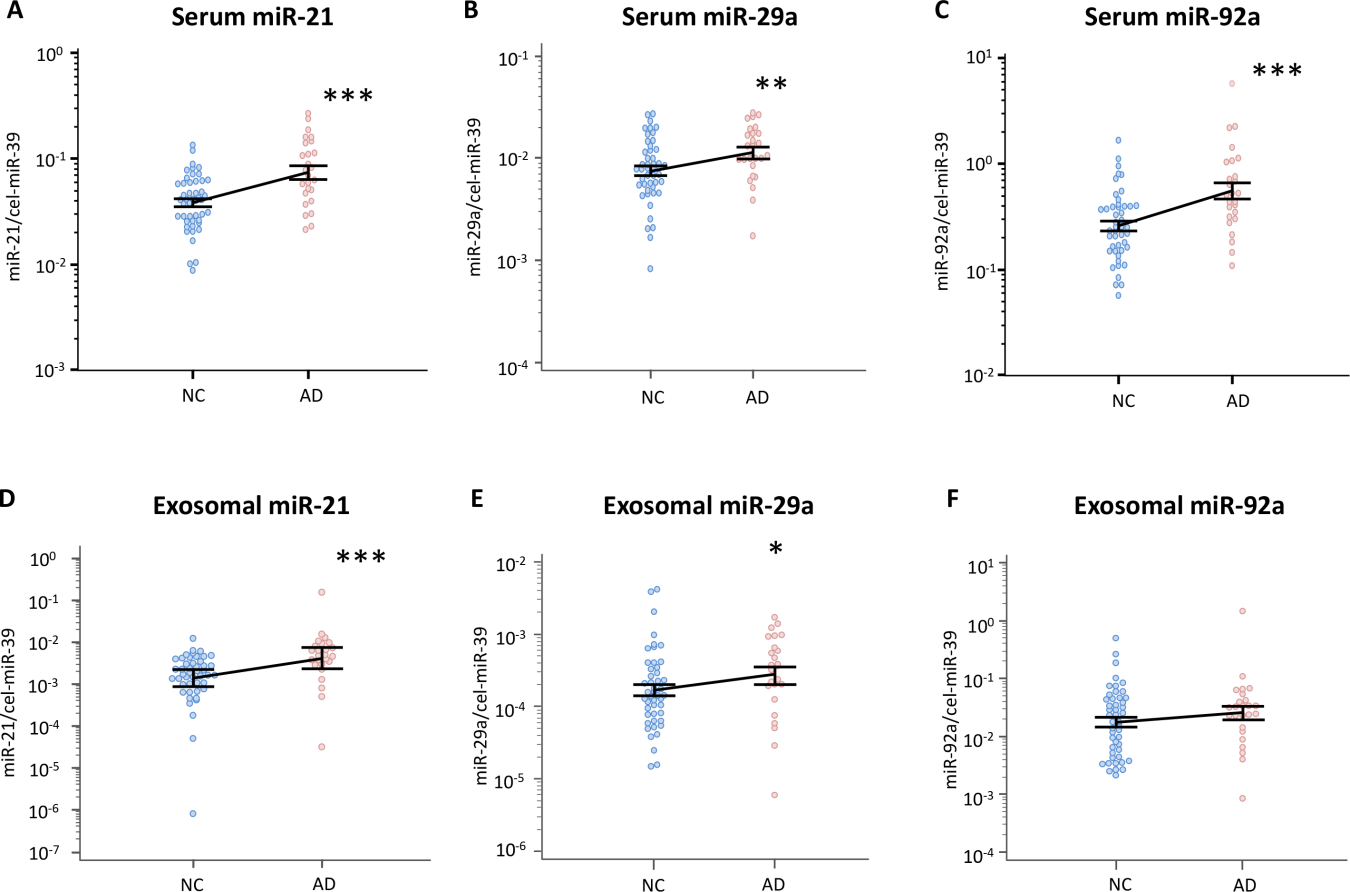
Serum or exosomal miRNAs levels in healthy controls and patients with colorectal adenomas. Plots are shown for serum levels of **(A)** miR-21, **(B)** miR-29a, **(C)** miR-92a, and exosomal **(D)** miR-21, **(E)** miR-29a and **(F)** miR-92a, in normal control subjects (NC; n = 47) and adenoma patients (AD; n = 26). The y-axis (log10 scale) represents relative expression of miRNAs normalized to cel-miR-39. All assays were carried out in duplicate. Data (means ± standard deviation) were analyzed by the Mann–Whitney U test (*P<0.05; **P<0.01; ***P<0.001).

Next, we investigated the expression levels of miR-21, miR-29a, miR-92a, and miR-135b in serum from healthy volunteers and patients with colorectal adenomas. Serum miR-21, miR-29a, miR-92a can be amplified and quantified; however, the expression levels of miR-135b were extremely low and could not be detected. Evaluating their clinical significance, the results indicated that serum miR-21, miR-29a, miR-92a in adenoma patients were significantly elevated vis-à-vis healthy volunteers (*P* = 0.0003, *P* = 0.0133 and *P* = 0.0005, respectively; **Fig 2**).

### Expression levels of miR-21 and miR-29a in exosomes from adenoma patients are significantly higher than in healthy volunteers

With regards to exosomal miRNAs, miR-21, miR-29a, miR-92a were successfully amplified and quantified, although miR-135b levels could not be detected (**Fig 2)**. The results indicated that exosomal miR-21 and miR-29a in adenoma patients were significantly elevated compared with that from healthy volunteers (*P* = 0.0002, and *P* = 0.05, respectively; **Fig 2**).

### Total serum miRNAs levels correlated with exosomal miRNAs levels

We investigated the correlation between total serum and exosomal miRNAs levels. We discovered that miR-21, miR-29a and miR-92a levels in serum were significantly correlated with those in exosomes, respectively (miR-21: rho = 0.542, *P* < 0.0001; miR-29a: rho = 0.322, *P* = 0.0059; miR-92a: rho = 0.310, *P* = 0.0081; **S2 Fig**).

### Total serum miR-21, miR-29a, miR-92a and exosomal miR-21 can identify patients with colonic adenomas (especially, high risk adenomas) with high accuracy

**Fig 3:**
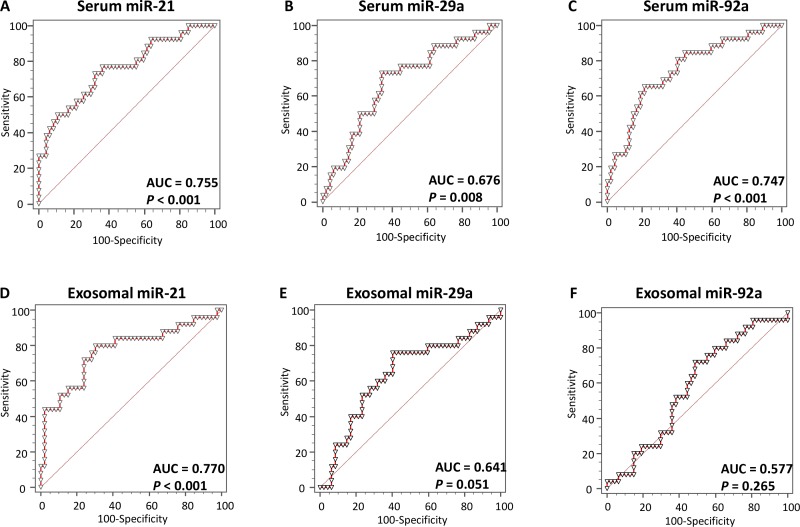
Receiver operating characteristics (ROC) curve analysis using serum or exosomal miRNAs levels to distinguish adenoma patients from healthy controls. **(A)** Serum miR-21 levels yielded an AUC value of 0.775 (95% CI = 0.640–0.848) in distinguishing adenoma patients from healthy controls; **(B)** Serum miR-29a levels yielded AUC values of 0.676 (95% CI = 0.556–0.781); **(C)** Serum miR-92a levels yielded AUC values of 0.747 (95% CI = 0.632–0.842); **(D)** Exosomal miR-21 levels yielded AUC values of 0.770 (95% CI = 0.654–0.861); **(E)** Exosomal miR-29a levels yielded AUC values of 0.641 (95% CI = 0.519–0.751); **(F)** Exosomal miR-92a levels yielded AUC values of 0.577 (95% CI = 0.455–0.693).

Next, we generated ROC curves to assess the potential usefulness of selected miRNAs in total serum and exosomes as potential non-invasive biomarkers for the early diagnosis of colorectal adenomas (**Fig 3**). Our ROC analyses revealed that serum miR-21, miR-29a, miR-92a and exosomal miR-21 levels significantly discriminated adenoma patients from control subjects, with AUC values of 0.755 (95% confidence interval (CI) = 0.640–0.848), 0.676 (95% CI = 0.556–0.781), 0.747 (95% CI = 0.632–0.842) and 0.770 (95% CI = 0.654–0.861) for serum miR-21, miR-29a, miR-92a and exosomal miR-21, respectively **(Fig 3**). Using cutoff values from the Youden’s index, the sensitivity and specificity of miR-21 were 73.1% and 68.1%, respectively, for identifying a patient with colorectal adenoma. Moreover, serum miR-29a yielded a sensitivity of 72.0% and a specificity of 66.0%; serum miR-92a yielded a sensitivity of 65.4% and a specificity of 78.7%; exosomal miR-21 demonstrated a sensitivity of 69.8% and a specificity of 80.0% to discriminate adenoma patients from healthy controls.

**Fig 4:**
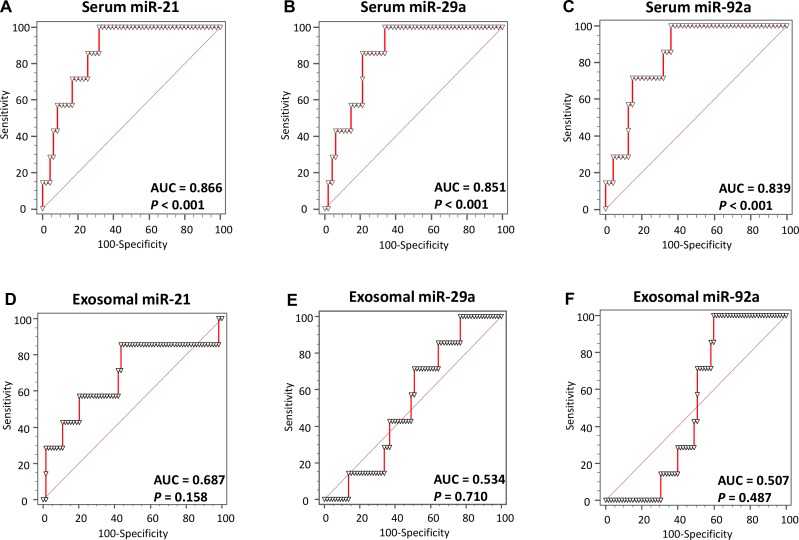
Receiver operating characteristics (ROC) curve analysis using serum or exosomal miRNA levels to distinguish advanced adenoma (>10 mm) patients from healthy controls. **(A)** Serum miR-21 levels yielded an AUC value of 0.775 (95% CI = 0.640–0.848) in distinguishing advanced adenoma patients from healthy controls; **(B)** Serum miR-29a levels yielded AUC values of 0.676 (95% CI = 0.556–0.781); **(C)** Serum miR-92a levels yielded AUC values of 0.747 (95% CI = 0.632–0.842); **(D)** Exosomal miR-21 levels yielded AUC values of 0.687 (95% CI = 0.566–0.792); **(E)** Exosomal miR-29a levels yielded AUC values of 0.534 (95% CI = 0.413–0.653); **(F)** Exosomal miR-92a levels yielded AUC values of 0.507 (95% CI = 0.393–0.634).

We further generated ROC curves to assess the potential of each miRNA as a non-invasive biomarker for the diagnosis of advanced, high-risk adenoma (>1.0 cm; **Fig 4**). Our ROC analyses revealed that the capacity of total serum miRNAs to discriminate between patients with advanced adenomas from healthy controls improved with higher AUC values up to around 85% (serum miR-21: AUC = 0.866; serum miR-29a: AUC = 0.851; serum miR-92a: AUC = 0.839; **Fig 4**), whereas the capacity of exosomal miRNAs for biomarker did not improve, and the statistical significance of results disappeared (**Fig 4**).

In addition, multivariable logistic regression analyses revealed that serum miR-21, miR-29a and miR-92a may be potential diagnostic biomarkers for the identification of patients with adenomas after adjustment for patient age and sex (**Table 2**). The OR for patients with serum miR-21 cut-off threshold values of >0.0484 associated adenomas was 5.24 (95% CI = 1.79–15.32), for cases with serum miR-29a of >0.0088 and with serum miR-92a of >0.398 associated with adenomas, they were 5.27 and 5.94, respectively (miR-29a: 95% CI = 1.46–14.4; miR-92a: 95% CI = 2.05–17.21; **Table 2**).

**Table 2:** Multivariable logistic analyses for serum miR-21 and miR-92a levels and various diagnostic factors in patients with adenoma.

Variables	OR (% CI *)	*p-*Value
Adenoma patients vs control subjects		
Age, >66 y vs ≤66 y^†^	0.61 (0.21–1.75)	0.36
Sex, male vs female	1.14 (0.39–3.26)	0.80
miR-21 in serum, > 0.0484 vs ≤0.0484^‡^	5.24 (1.79–15.32)	0.0025
Adenoma patients vs control subjects		
Age, >66 y vs ≤66 y^†^	0.88 (0.30–2.53)	0.81
Sex, male vs female	0.79 (0.25–2.40)	0.67
miR-29a in serum, >0.0088 vs ≤0.0088^‡^	5.27 (1.68–16.54)	0.0043
Adenoma patients vs control subjects		
Age, >66 y vs ≤66 y^†^	0.73 (0.25–2.10)	0.56
Sex, male vs female	1.14 (0.39–3.31)	0.80
miR-92a in serum, >0.398 vs ≤0.398^‡^	5.94 (2.05–17.21)	0.001

CI * = confidence interval; OR = odds ratio.

^†^ Median age was 66 years respectively.

^‡^ The cutoff values of serum miR-21, miR-29a and miR-92a in adenoma patients vs. control subjects were derived by receiver operating characteristic curves with Youden’s index.

### Relationship between total serum and exosomal microRNA levels and clinical features of patients with adenomatous polyps

We found that total serum miR-21, miR-29a and miR-92a levels were significantly correlated with diameter at the equator of adenomatous polyps (miR-21: rho = 0.422, *P* = 0.0002; miR-29a: rho = 0.305, *P* = 0.0092; miR-92a: rho = 0.413, *P* = 0.0003; **S3 Fig**), while as for exosomal microRNAs, only miR-21 levels were significantly correlated with diameter at the equator of adenomatous polyps (rho = 0.435, *P* = 0.0001; **S3 Fig**). Additionally, significant correlations between the polyp count in the colorectum and microRNA levels were identified (serum miR-21: rho = 0.379, *P* = 0.001; serum miR-29a: rho = 0.297, *P* = 0.012; serum miR-92a: rho = 0.361, *P* = 0.0019; exosomal miR-21: rho = 0.439, *P* = 0.0001; exosomal miR-29a: rho = 0.261, *P* = 0.027; **S4 Fig**). Collectively, although total serum and exosomal miRNAs in adenoma patients might be derived from the adenoma itself, our data suggest that total serum miRNAs levels are a more accurate reflection of the disease status of adenomas in colorectum, compared to the exosomal miRNA levels.

## Discussion

Most methods for the identification of diagnostic circulating miRNAs have largely focused on miRNAs that are frequently overexpressed in cancers [11, 29]. Ironically however, only a few miRNAs that are abundantly expressed in cancer cells are detectable in blood [30], and nearly 30% of secreted miRNAs do not affect the cellular expression profiles witnessed in specific cancer cells [31]. These data highlight the need for more careful evaluation of the paradigm whether these miRNAs are retained in the cell or are released into the systemic circulation to provide a robust substrate for a noninvasive diagnostic strategy [17]. A comprehensive literature search identified only four candidate miRNAs that afforded discriminative biomarker potential for identifying patients with colorectal neoplasia (adenomas and cancers) based upon their overexpression in these patient’s tissues and blood circulation.

Our study validated four miRNAs (miR-21, miR-29a, miR-92a, and miR-135b) as truly upregulated in colorectal polyps and cancers, which is consistent with previously published reports listed in **S1 Table**. Recent systematic reviews have demonstrated that MiR-21 participates in several steps of colorectal tumorigenesis by regulation of MAPK pathway and associating with WNT/β-Catenin signaling [32]. In addition, miR-135a/b and miR-17-92a cluster also activate the WNT signaling pathway via suppression of APC or E2F1, respectively [32]. These evidences clearly indicate the functional role of miRNAs included in this study to be involved in the early phases of multi-step colorectal carcinogenesis via regulation of WNT/β-Catenin, and MAPK signal pathways. Three of the four miRNAs (miR-21, miR-92a, and miR-135b) demonstrated a stepwise increase in their expression in accordance with the normal-adenoma-carcinoma sequence. Furthermore, our study provided a unique advantage since we compared total serum miRNAs with exosomal miRNAs extracted from the same samples to assess their comparative biomarker potential for identifying adenoma patients from healthy controls.

Standardization of associated methodologies including serum/plasma sample storage, RNA extraction, and quantification of circulating miRNAs, is urgently needed for developing clinically-relevant biomarkers [33]. Our study, for the first time, demonstrated interesting findings when we performed head-to-head comparison between miRNA expression levels between total serum and exosomal fractions, and demonstrated that serum miRNAs are superior diagnostic biomarkers for adenoma patients. Taylor et al. previously demonstrated that miRNA profiles in circulating tumor-derived exosomes and the matched primary tumor tissues were comparable [34], suggesting the possibility that miRNA profiling of circulating exosomes maybe an option in future studies. In addition, Tanaka et al. showed that miR-21 expression is higher in exosomes than total serum [25], indicating that the majority of the circulating miR-21 signal may be contributed from exosomes. These evidences are consistent with our 3 results described below. First, both total serum and exosomal miR-21 are significantly associated with the size and adenoma count in the colorectum. Second, significant correlation was recognized between total serum and exosomal miR-21 levels. Third, diagnostic potential of both total serum and exosomal miR-21 for adenoma patients was quite comparable. Taken together, these data highlight that even though we analyzed total serum and exosomal miRNAs, miR-21 remains an important biomarker for accurately discriminating adenoma patients from healthy subjects. MiR-21 is one of the most abundantly expressed miRNAs in CRC and is also over-expressed in other diseases such as several cancers and chronic inflammation disease [35]. Overall, miR-21 is not a specific diagnostic marker in CRC; it requires to be combined with other microRNAs for enhanced specificity.

In contrast, quantification of total serum miRNAs (miR-29a and miR-92a) is superior in discriminating adenoma patients from healthy controls. Most miRNAs are enclosed in microvesicles, primarily exosomes in circulation [36]. However, some exist extracellularly either by binding to proteins (Argonaute2), vesicles, and lipoprotein particles [37, 38], or making the hairpin-loop structure that protects them from endonuclease and enzyme degradation [39]. In other words, with the exception for exosomal miRNAs, secreted miRNAs in circulation might affect miRNA-based diagnostic potential to some extent. In addition, exosomes are usually isolated from serum/plasma during an overnight ultracentrifugation step [40, 41], which is labor-intensive, time-consuming and also requires expensive laboratory equipment, making it less practical for clinical applications. Taken together, accumulating facts strongly recommend that using total serum miRNAs may still be preferable than exosomal miRNAs as diagnostic markers in colorectal neoplasia.

This study also demonstrated the potential role of total serum miR-21, miR-29a and miR-92a in the early detection of colorectal adenomas and cancers. This is supported by markedly high AUC values of 0.755, 0.676, 0.747 and 0.770, respectively, derived from comparisons between adenoma patients and healthy control subjects. Even more important from a clinical perspective, to identify large, high-risk adenomas that are considered target lesions for CRC screening [4], total serum miR-21, miR-29a and miR-92a expression levels demonstrated very high AUC values of 0.886, 0.851 and 0.839, respectively, which associated with high sensitivity and specificity, for discerning patients with large adenomatous polyps from healthy control subjects. Additionally, the ORs for patients with high levels of total serum miR-21, miR-29a and miR-92a expression associated with adenomatous polyps that were >10 mm in diameter were 5.24 (95% CI = 1.79–15.32), 5.27 (95% CI = 1.68–16.54) and 5.94 (95% CI = 2.05–17.21), which are extraordinary performance levels for non-invasive biomarkers compared with recently reported data for a positive first-guaiac FOBT (OR [95% CI] = 3.1 [1.86–5.18]) [42]. Thus, our study highlights that miR-21, miR-29a and miR-92a expression levels in total serum can be used as promising non-invasive biomarkers for the early detection of CRC, as well as for the identification of clinically meaningful, high-risk adenomatous polyps—a critical target lesion for any CRC screening strategy.

In conclusion, our results revealed that although total serum miRNAs could serve as non-invasive biomarkers for the early detection of CRC, exosomal miRNAs were unfavorable for the identification of high-risk adenomatous polyps. This study provides compelling evidence for the potential usefulness of serum miR-21, miR-29a and miR-92a as non-invasive screening tools in patients with adenomas, a concept that can be incorporated into routine clinical practice in the not-so-distant future pending validation in large-scale prospective trials.

## Supporting Information

S1 Fig The flowchart illustrates the procedure for extracting serum and exosomal miRNAs from same patient specimens. Total 500 μL of serum were divided into two specimens to elute miRNAs in serum and in exosome from each specimen, respectively.
(PDF)

S2 Fig Scatter plots showing the correlation between exosomal miRNA levels and serum miRNA levels. Positive correlation was noticed between **(A)** total serum and exosomal miR-21 levels; **(B)** total serum and exosomal miR-29a levels; **(C)** total serum and exosomal miR-92a levels. Data were analyzed using the Spearman’s correlation test.
(PDF)

S3 Fig Scatter plots showing the correlation between serum or exosomal miRNAs levels and maximum diameter of adenomas. Positive correlation between maximum diameter of adenomas and **(A)** serum miR-21 levels (rho = 0.422; *P* = 0.0002); **(B)** serum miR-29a levels (rho = 0.305; *P* = 0.0092); **(C)** serum miR-92a levels (rho = 0.413; *P* = 0.0003); **(D)** exosomal miR-21 levels (rho = 0.435; *P* = 0.0001). No significant correlation between maximum diameter of adenomas and **(E)** exosomal miR-29a levels (rho = 0.223; *P* = 0.0593); **(F)** exosomal miR-92a levels (rho = 0.108; *P =* 0.3654). Data were analyzed using the Spearman correlation test.
(PDF)

S4 Fig Scatter plots showing the correlation between serum or exosomal miRNAs levels and adenoma number in the entire colorectum. Positive correlation between adenoma counts in the colorectum and **(A)** serum miR-21 levels (rho = 0.379; *P* = 0.001); **(B)** serum miR-29a levels (rho = 0.297; *P* = 0.012); **(C)** serum miR-92a levels (rho = 0.361; *P* = 0.0019); **(D)** exosomal miR-21 levels (rho = 0.439; *P*<0.0001); **(E)** exosomal miR-29a levels (rho = 0.261; *P =* 0.0270). No significant correlation between adenoma counts in the colorectum and **(F)** exosomal miR-92a levels (rho = 0.142; *P* = 0.2335). Data were analyzed using the Spearman’s correlation test.
(PDF)

S1 Table Candidate microRNAs reported to be overexpressed in both CRC tissues and circulation from CRC patients. (DOCX)

S2 Table Patient characteristics. (DOCX)
