# Supplementary figures and images for: Diagnostic Potential of Cell-Free and Exosomal MicroRNAs in the Identification of Patients with High-Risk Colorectal Adenomas
Source: PLoS One. 2016 Oct 19;11(10):e0160722. doi: 10.1371/journal.pone.0160722 (PMC5070810; doi:10.1371/journal.pone.0160722)

S1 Fig.

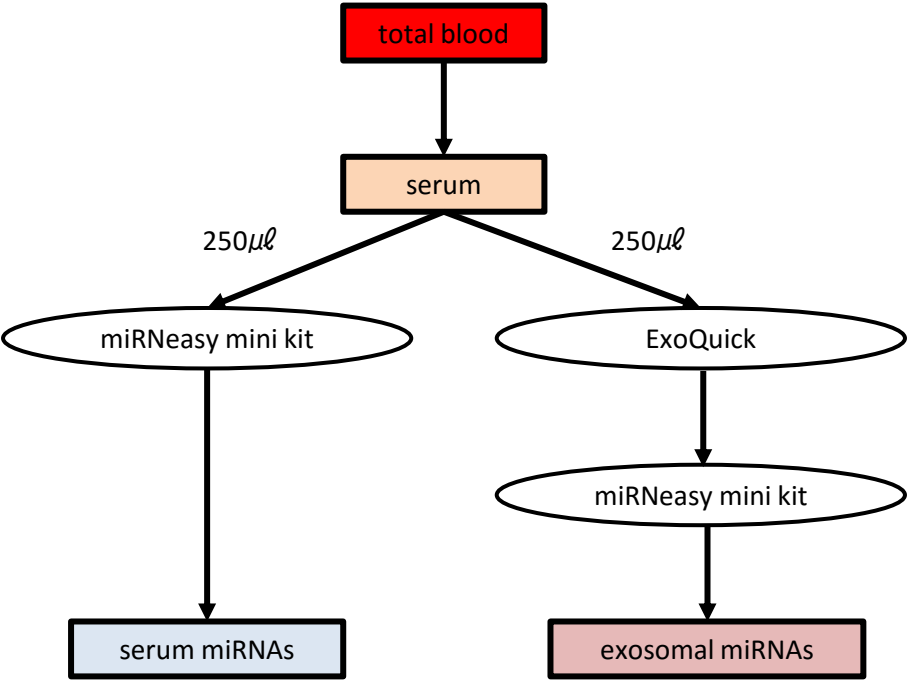

Supplement: S1 Fig — Total 500 μL of serum were divided into two specimens to elute miRNAs in serum and in exosome from each specimen, respectively. (PDF) [file pone.0160722.s001.pdf]

S2 Fig.

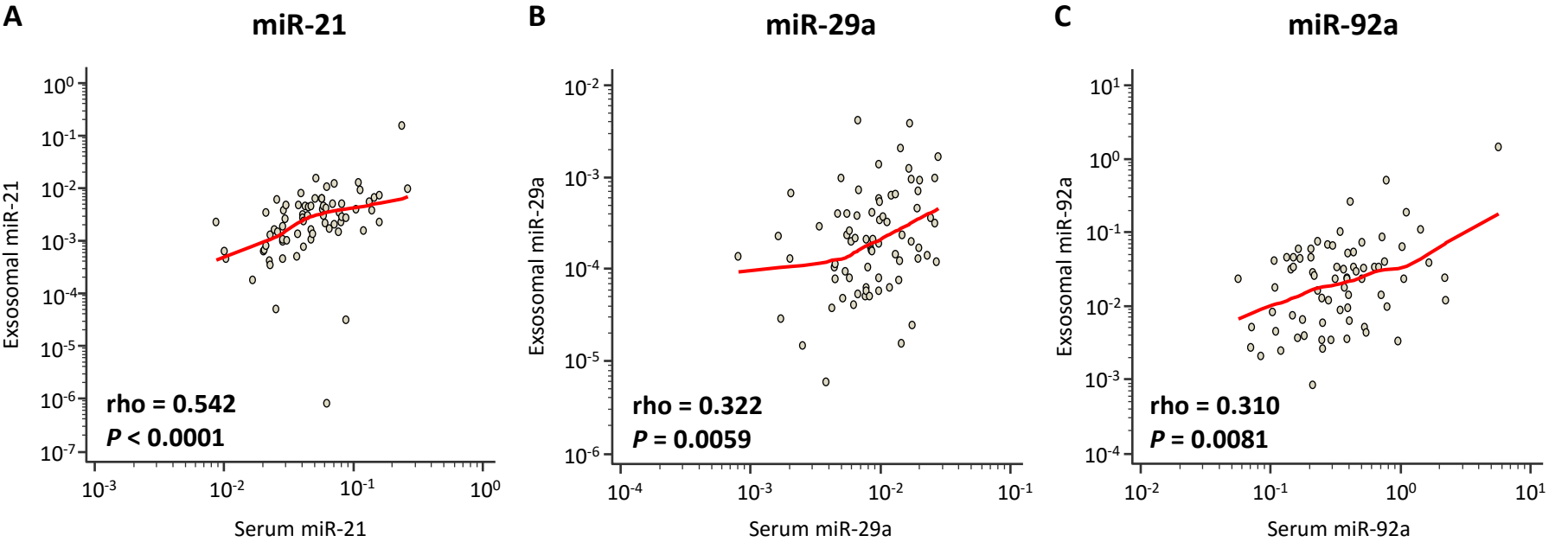

Supplement: S2 Fig — Positive correlation was noticed between (A) total serum and exosomal miR-21 levels; (B) total serum and exosomal miR-29a levels; (C) total serum and exosomal miR-92a levels. Data were analyzed using the Spearman’s correlation test. (PDF) [file pone.0160722.s002.pdf]

**S3 Fig.**

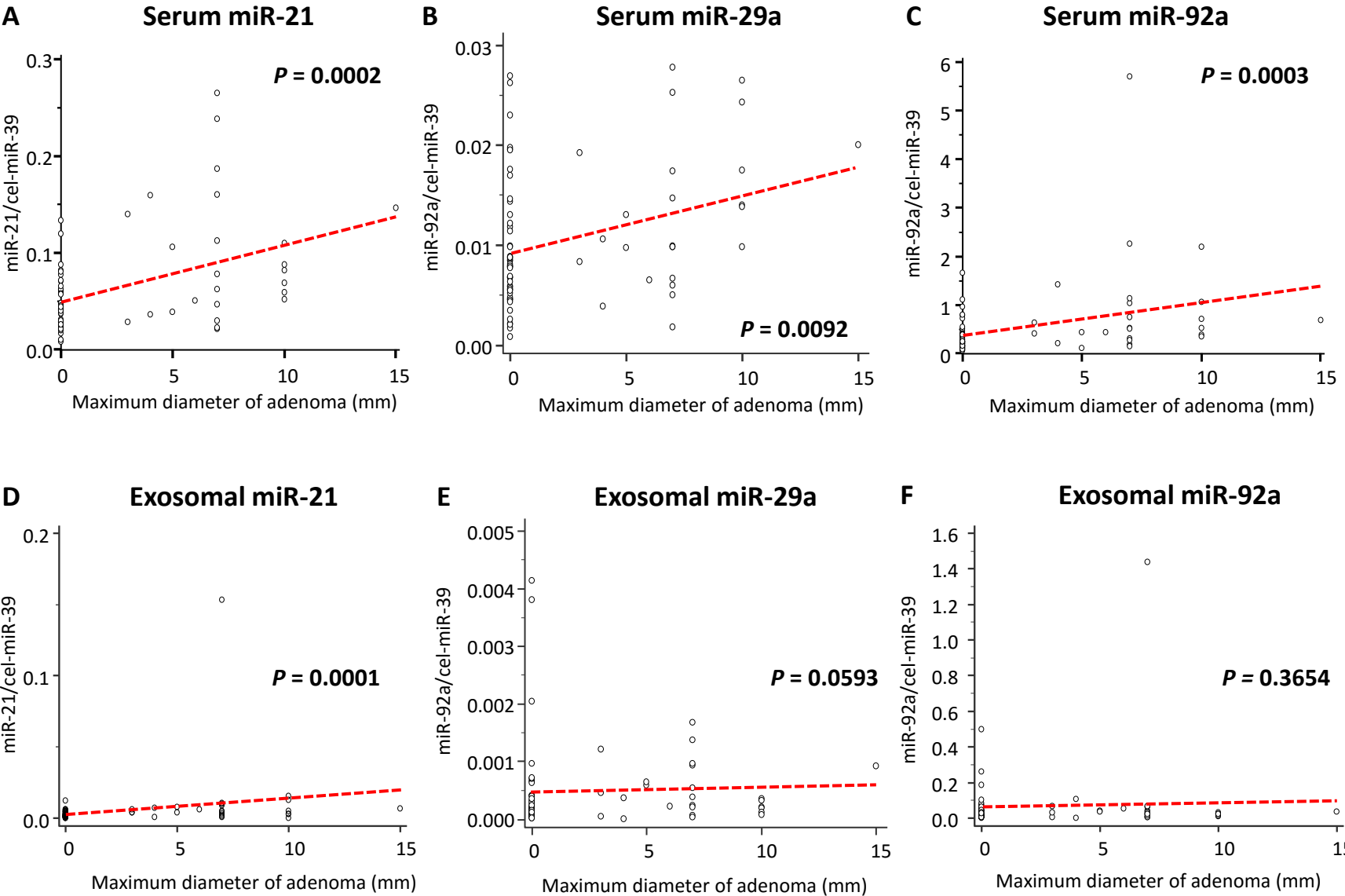

Supplement: S3 Fig — Positive correlation between maximum diameter of adenomas and (A) serum miR-21 levels (rho = 0.422; P = 0.0002); (B) serum miR-29a levels (rho = 0.305; P = 0.0092); (C) serum miR-92a levels (rho = 0.413; P = 0.0003); (D) exosomal miR-21 levels (rho = 0.435; P = 0.0001). No significant correlation between maximum diameter of adenomas and (E) exosomal miR-29a levels (rho = 0.223; P = 0.0593); (F) exosomal miR-92a levels (rho = 0.108; P = 0.3654). Data were analyzed using the Spearman correlation test. (PDF) [file pone.0160722.s003.pdf]

S4 Fig.

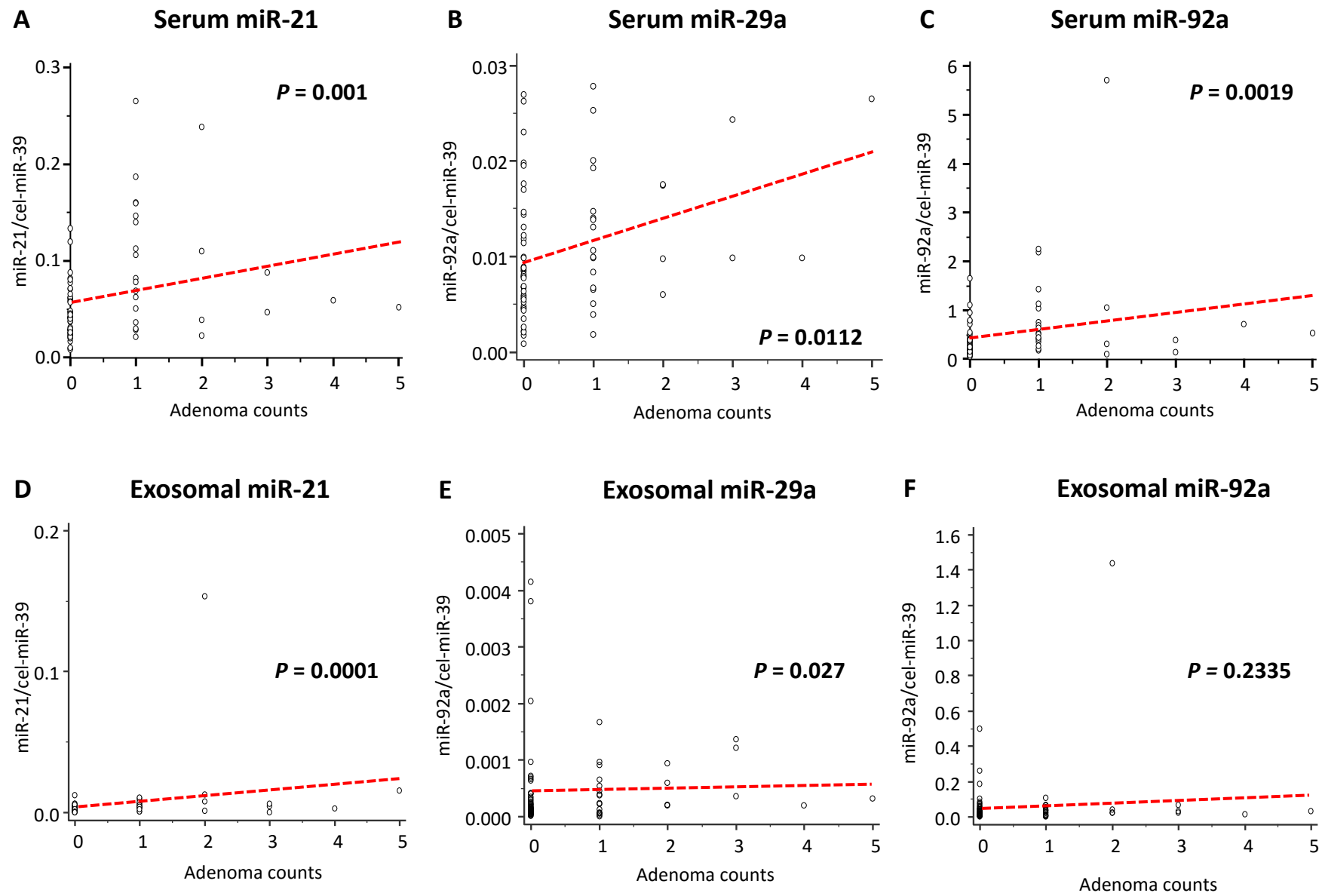

Supplement: S4 Fig — Positive correlation between adenoma counts in the colorectum and (A) serum miR-21 levels (rho = 0.379; P = 0.001); (B) serum miR-29a levels (rho = 0.297; P = 0.012); (C) serum miR-92a levels (rho = 0.361; P = 0.0019); (D) exosomal miR-21 levels (rho = 0.439; P<0.0001); (E) exosomal miR-29a levels (rho = 0.261; P = 0.0270). No significant correlation between adenoma counts in the colorectum and (F) exosomal miR-92a levels (rho = 0.142; P = 0.2335). Data were analyzed using the Spearman’s correlation test. (PDF) [file pone.0160722.s004.pdf]
