# Supplementary material for: Diagnostic Potential of Cell-Free and Exosomal MicroRNAs in the Identification of Patients with High-Risk Colorectal Adenomas
Source: PLoS One. 2016 Oct 19;11(10):e0160722. doi: 10.1371/journal.pone.0160722 (PMC5070810; doi:10.1371/journal.pone.0160722)
Supplement: S1 Table — (DOCX) [file pone.0160722.s005.docx]

**S1 Table**

| **Candidate microRNAs** | **miRNAs level and clinical significance in tissues** | **miRNAs level and clinical significance in circulation** |
| --- | --- | --- |
| ***miR-21*** | Most extensively investigated oncogenic microRNA whose expression is frequently up-regulated in CRC. Overexpression in tissues correlates with recurrence ,poor prognosis and chemo resistance (1-5) | Increased in CRC compared with controls (6) |
|  |  | Increased in CRC and advanced adenomas compared with controls (7) |
|  |  | Increased in CRC and advanced adenomas compared with controls (8) |
|  |  | Correlation with the recurrence and mortality of CRC patients (9) |
|  |  |  |
| ***miR-29a*** | Increased in CRC tissues and overexpression in tissues correlates with recurrence and poor prognosis.(10-12) | Increased in CRC and advanced adenomas compared with controls (13) |
|  |  | Increased in CRC compared with controls (14) |
|  |  | Increased in CRC with liver metastasis compared with non-metastatic CRC (15) |
|  |  | Increased in stage III CRC compared with controls (16) |
| ***miR-92a*** | Increased in CRC tissues and overexpression in tissues correlates with tumor metastasis and poor prognosis.(17-20) | Increased in CRC patients; decrease in post-operative samples (21) |
|  |  | Increased in CRC and advanced adenomas compared with controls (13) |
|  |  | Increased in CRC and advanced adenomas compared with controls (7) |
| ***miR-135b*** | Increased in CRC tissues, which correlates with clinical stage, liver metastasis, and both disease-free survival (DFS) and cancer-specific survival of patients (22-24) | Increased in CRC compared with controls (21) |

**References of S1 Table**

1. Schetter AJ, Leung SY, Sohn JJ, Zanetti KA, Bowman ED, Yanaihara N, et al. MicroRNA expression profiles associated with prognosis and therapeutic outcome in colon adenocarcinoma. JAMA 2008;299(4):425-36.

2. Kulda V, Pesta M, Topolcan O, Liska V, Treska V, Sutnar A, et al. Relevance of miR-21 and miR-143 expression in tissue samples of colorectal carcinoma and its liver metastases. Cancer Genet Cytogenet 2010;200(2):154-60.

3. Shibuya H, Iinuma H, Shimada R, Horiuchi A, Watanabe T. Clinicopathological and prognostic value of microRNA-21 and microRNA-155 in colorectal cancer. Oncology 2010;79(3-4):313-20.

4. Schetter AJ, Nguyen GH, Bowman ED, Mathe EA, Yuen ST, Hawkes JE, et al. Association of inflammation-related and microRNA gene expression with cancer-specific mortality of colon adenocarcinoma. Clin Cancer Res 2009;15(18):5878-87.

5. Nielsen BS, Jorgensen S, Fog JU, Sokilde R, Christensen IJ, Hansen U, et al. High levels of microRNA-21 in the stroma of colorectal cancers predict short disease-free survival in stage II colon cancer patients. Clin Exp Metastasis 2011;28(1):27-38.

6. Kanaan Z, Rai SN, Eichenberger MR, Roberts H, Keskey B, Pan J, et al. Plasma miR-21: a potential diagnostic marker of colorectal cancer. Ann Surg 2012;256(3):544-51.

7. Liu GH, Zhou ZG, Chen R, Wang MJ, Zhou B, Li Y, et al. Serum miR-21 and miR-92a as biomarkers in the diagnosis and prognosis of colorectal cancer. Tumour Biol 2013;34(4):2175-81.

8. Toiyama Y, Takahashi M, Hur K, Nagasaka T, Tanaka K, Inoue Y, et al. Serum miR-21 as a diagnostic and prognostic biomarker in colorectal cancer. J Natl Cancer Inst 2013;105(12):849-59.

9. Menendez P, Padilla D, Villarejo P, Palomino T, Nieto P, Menendez JM, et al. Prognostic implications of serum microRNA-21 in colorectal cancer. J Surg Oncol 2013;108(6):369-73.

10. Fu J, Tang W, Du P, Wang G, Chen W, Li J, et al. Identifying microRNA-mRNA regulatory network in colorectal cancer by a combination of expression profile and bioinformatics analysis. BMC Syst Biol 2012;6:68.

11. Weissmann-Brenner A, Kushnir M, Lithwick Yanai G, Aharonov R, Gibori H, Purim O, et al. Tumor microRNA-29a expression and the risk of recurrence in stage II colon cancer. Int J Oncol 2012;40(6):2097-103.

12. Kuo TY, Hsi E, Yang IP, Tsai PC, Wang JY, Juo SH. Computational analysis of mRNA expression profiles identifies microRNA-29a/c as predictor of colorectal cancer early recurrence. PLoS One 2012;7(2):e31587.

13. Huang Z, Huang D, Ni S, Peng Z, Sheng W, Du X. Plasma microRNAs are promising novel biomarkers for early detection of colorectal cancer. Int J Cancer 2010;127(1):118-26.

14. Giraldez MD, Lozano JJ, Ramirez G, Hijona E, Bujanda L, Castells A, et al. Circulating microRNAs as biomarkers of colorectal cancer: results from a genome-wide profiling and validation study. Clin Gastroenterol Hepatol 2013;11(6):681-8 e3.

15. Wang LG, Gu J. Serum microRNA-29a is a promising novel marker for early detection of colorectal liver metastasis. Cancer Epidemiol 2012;36(1):e61-7.

16. Brunet Vega A, Pericay C, Moya I, Ferrer A, Dotor E, Pisa A, et al. microRNA expression profile in stage III colorectal cancer: circulating miR-18a and miR-29a as promising biomarkers. Oncol Rep 2013;30(1):320-6.

17. Schee K, Boye K, Abrahamsen TW, Fodstad O, Flatmark K. Clinical relevance of microRNA miR-21, miR-31, miR-92a, miR-101, miR-106a and miR-145 in colorectal cancer. BMC Cancer 2012;12:505.

18. Wu CW, Ng SS, Dong YJ, Ng SC, Leung WW, Lee CW, et al. Detection of miR-92a and miR-21 in stool samples as potential screening biomarkers for colorectal cancer and polyps. Gut 2012;61(5):739-45.

19. Tsuchida A, Ohno S, Wu W, Borjigin N, Fujita K, Aoki T, et al. miR-92 is a key oncogenic component of the miR-17-92 cluster in colon cancer. Cancer Sci 2011;102(12):2264-71.

20. Yu G, Tang JQ, Tian ML, Li H, Wang X, Wu T, et al. Prognostic values of the miR-17-92 cluster and its paralogs in colon cancer. J Surg Oncol 2012;106(3):232-7.

21. Ng EK, Chong WW, Jin H, Lam EK, Shin VY, Yu J, et al. Differential expression of microRNAs in plasma of patients with colorectal cancer: a potential marker for colorectal cancer screening. Gut 2009;58(10):1375-81.

22. Xu XM, Qian JC, Deng ZL, Cai Z, Tang T, Wang P, et al. Expression of miR-21, miR-31, miR-96 and miR-135b is correlated with the clinical parameters of colorectal cancer. Oncol Lett 2012;4(2):339-345.

23. Faltejskova P, Svoboda M, Srutova K, Mlcochova J, Besse A, Nekvindova J, et al. Identification and functional screening of microRNAs highly deregulated in colorectal cancer. J Cell Mol Med 2012;16(11):2655-66.

24. Gaedcke J, Grade M, Camps J, Sokilde R, Kaczkowski B, Schetter AJ, et al. The rectal cancer microRNAome--microRNA expression in rectal cancer and matched normal mucosa. Clin Cancer Res 2012;18(18):4919-30.
