# Supplementary material for: Diagnostic Potential of Cell-Free and Exosomal MicroRNAs in the Identification of Patients with High-Risk Colorectal Adenomas
Source: PLoS One. 2016 Oct 19;11(10):e0160722. doi: 10.1371/journal.pone.0160722 (PMC5070810; doi:10.1371/journal.pone.0160722)
Supplement: S2 Table — (DOCX) [file pone.0160722.s006.docx]

| **S2 Table** | | | |
| --- | --- | --- | --- |
|  | **Adenoma patients** | **Healthy controls** | ***P-value*** |
| Characteristics | n=26 | n=47 |  |
|  |  |  |  |
| Age(years) |  |  |  |
| Mean±SD | 66±11.8 | 63 ±16.3 | ns |
| Gender |  |  |  |
| Male | 15 | 23 | ns |
| Female | 11 | 24 |  |
| SD: standard deviation; ns: not significant. | | | |
